# Supplementary material for: Structural insight into molecular mechanism of poly(ethylene terephthalate) degradation
Source: Nat Commun. 2018 Jan 26;9:382. doi: 10.1038/s41467-018-02881-1 (PMC5785972; doi:10.1038/s41467-018-02881-1)
Supplement: Supplementary file 1 — Supplementary Information [file 41467_2018_2881_MOESM1_ESM.pdf]

Supplementary information

Structural insight into molecular mechanism of poly(ethylene terephthalate) degradation

Seongjoon Joo, In Jin Cho, Hogyun Seo, Hyeoncheol Francis Son, Hye-Young Sagong, Tae Joo Shin, So Young Choi, Sang Yup Lee* & Kyung-Jin Kim*

*Correspondence should be addressed to: Email: kkim@knu.ac.kr
leesy@kaist.ac.kr

This PDF file includes:

Supplementary Figures 1 to 6.

Supplementary Tables 1 and 2

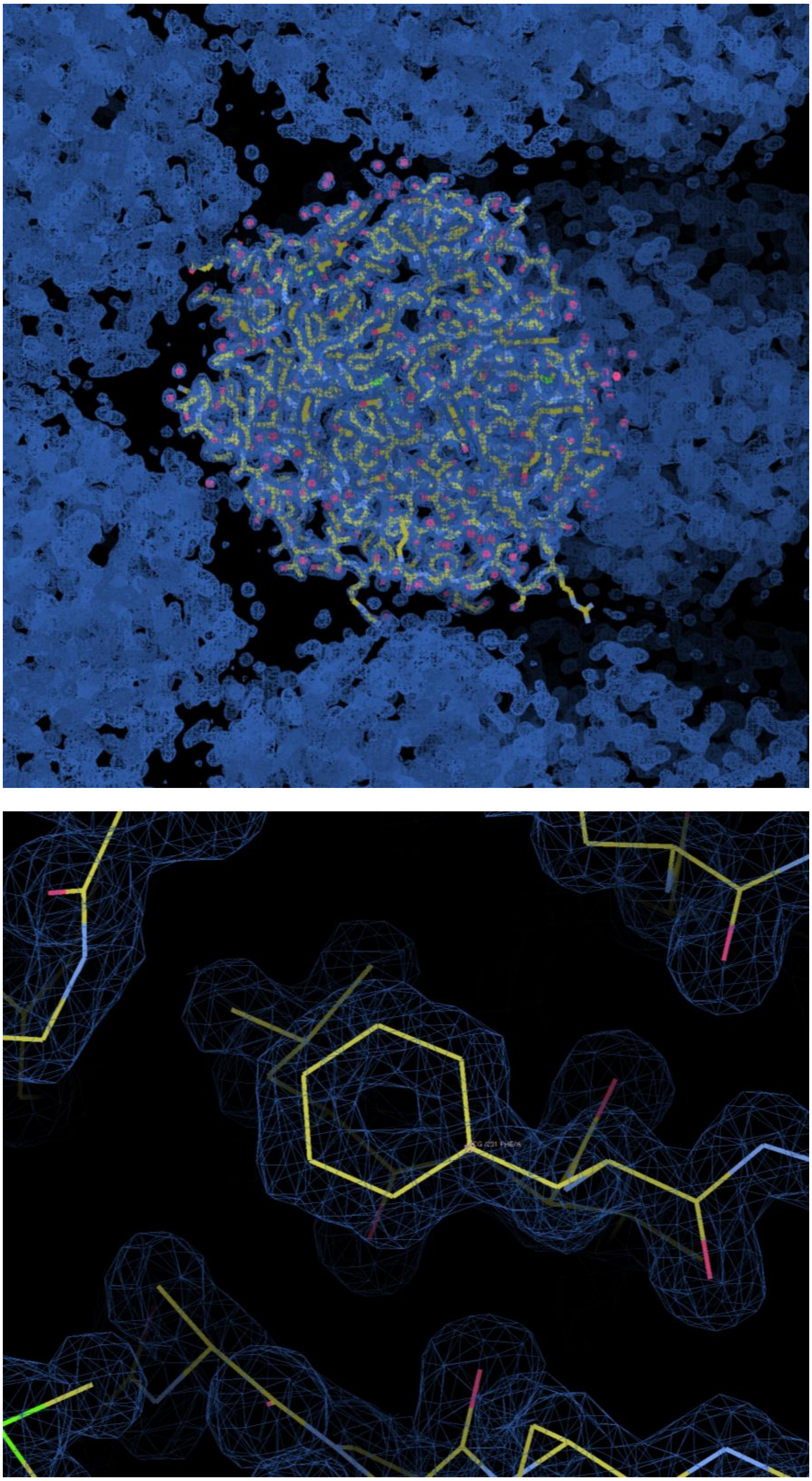

Supplementary Figure 1. The electron density map of *Is*PETase. The figures were generated by *WinCoot*. The $2F_0$-F_c map contoured at 1.5 σ is shown as blue mesh. The refined atomic model of *Is*PETase are shown as line with different color scheme by atoms; yellow-carbon, red-oxygen, blue-nitrogen, and green-sulfur. No crystallographic symmetry intimates the monomeric structure of *Is*PETase (above). The map represents the high resolution of 1.5 Å of *Is*PETase (below).

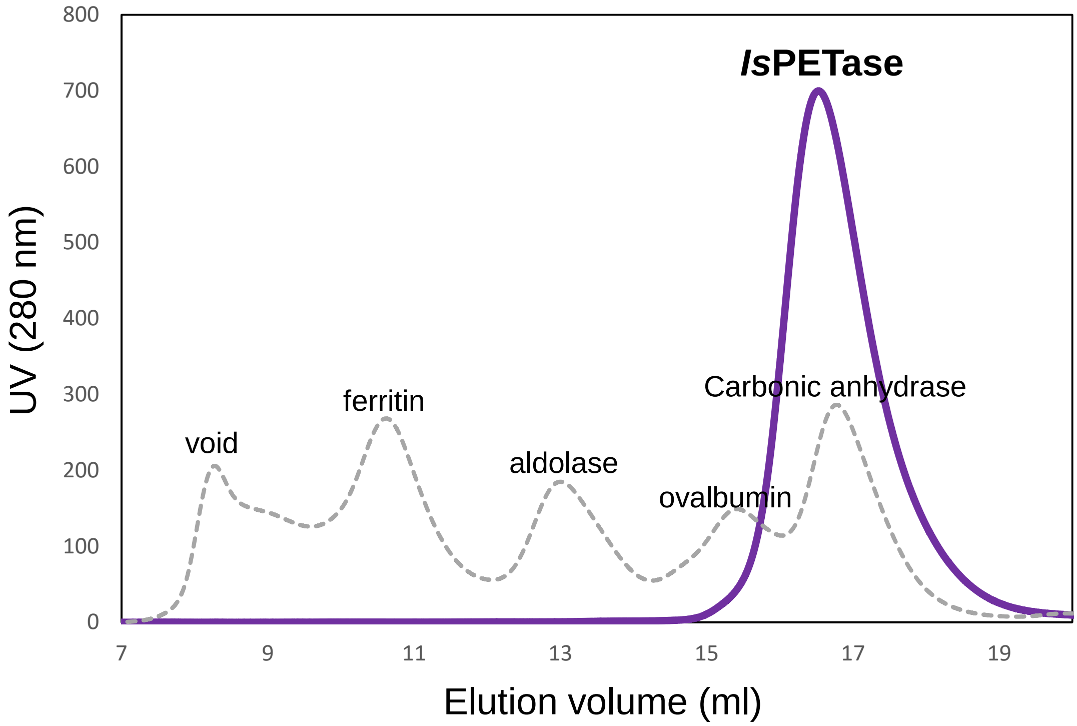

Supplementary Figure 2. Size-exclusion chromatographic analysis of *Is*PETase. A elution pick corresponding to a monomeric state of *Is*PETase in Size-exclusion chromatographic analysis. For precise analysis of the molecular weight, standard samples of ferritin (440kDa), aldolase (158kDa), ovalbumin (44kDa), and carbonic anhydrase (29kDa) are used for calibration and labelled.

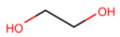

EG

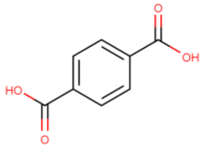

TPA

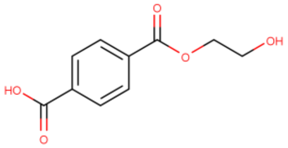

MHET

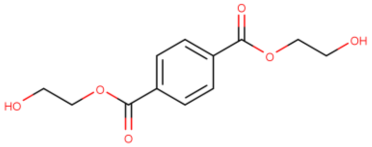

BHET

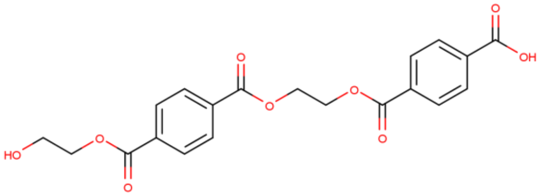

(MHET)$_2$

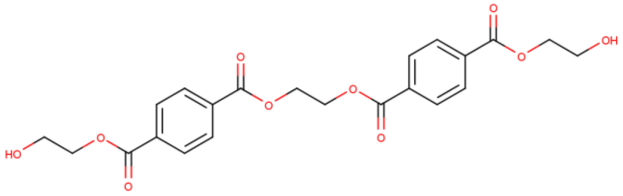

2-HE(MHET)$_2$

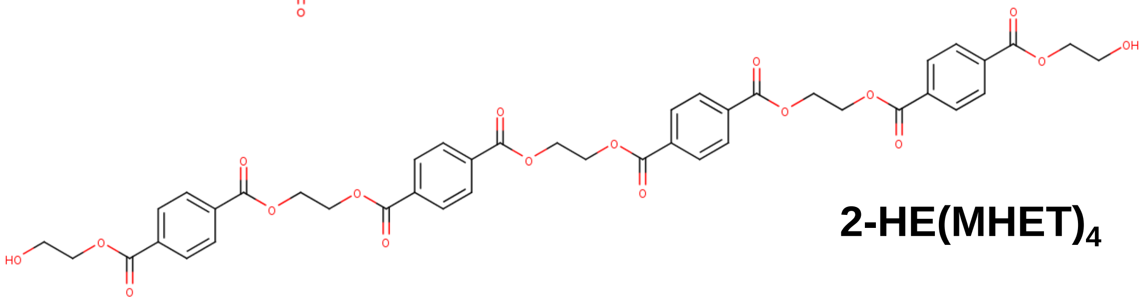

2-HE(MHET)$_4$

Supplementary Figure 3. Chemical structures of the PET-related molecules used in this study.

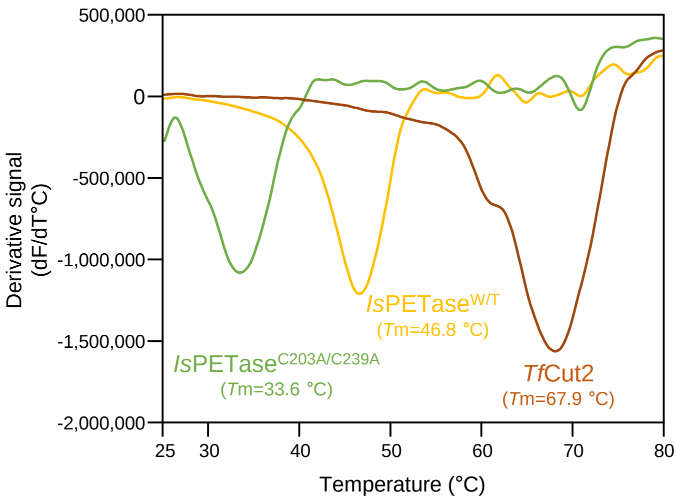

pH 7

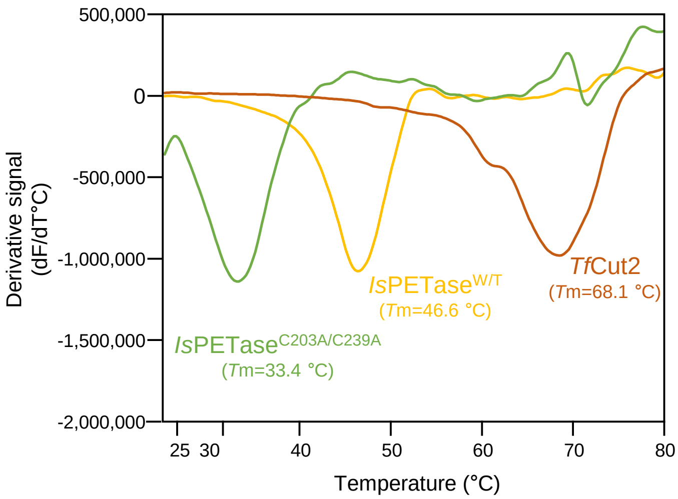

pH 9

Supplementary Figure 4. Thermal stability measurements of *Is*PETase$^{W/T}$, *Is*PETase$^{C203A/C239A}$, and *Tf*Cut2. The *Tm* values at pH 7 and 9 of *Is*PETase$^{W/T}$, *Is*PETase$^{C203A/C239A}$, and *Tf*Cut2 are displayed.

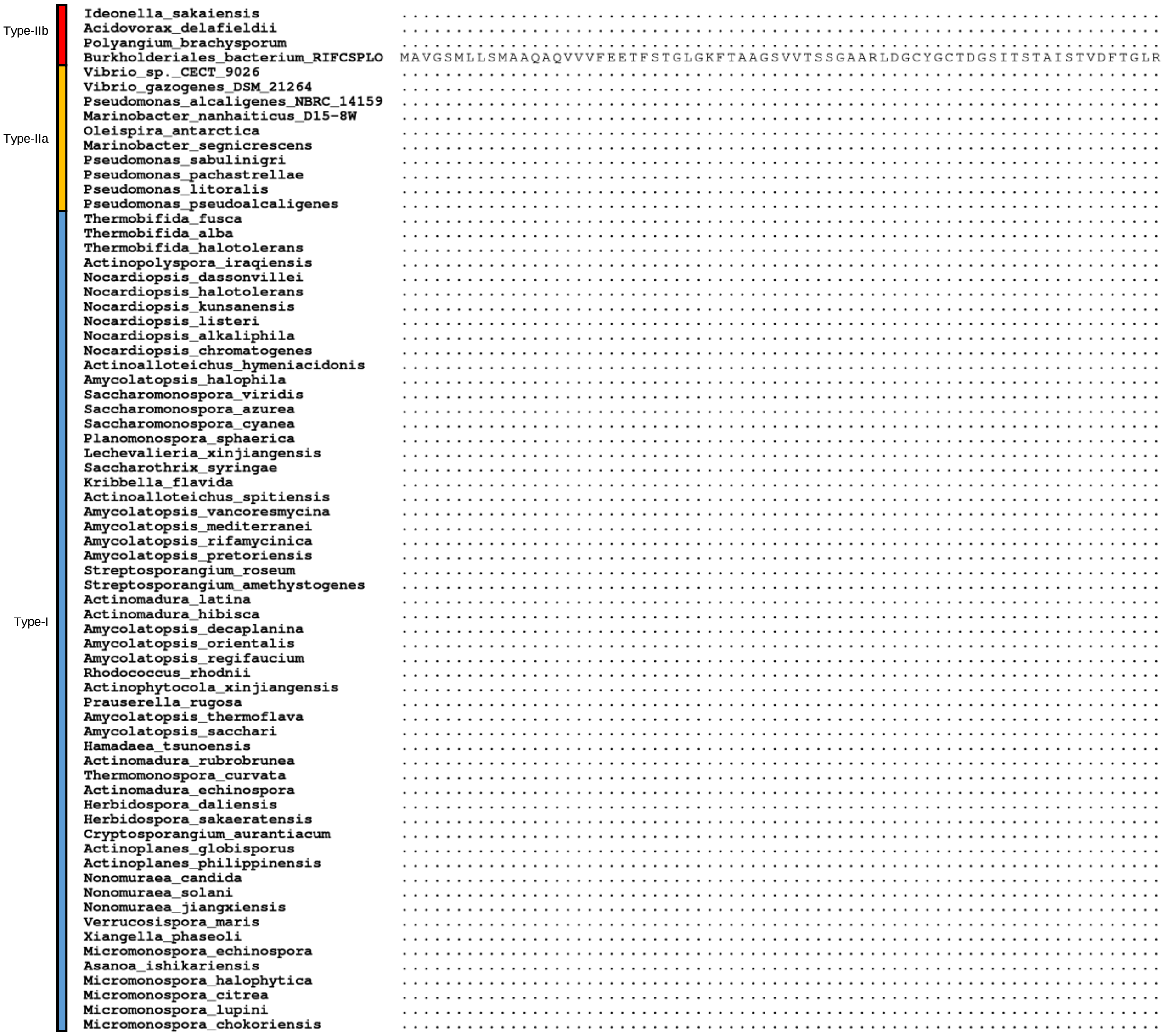

Supplementary Figure 5. Amino acid sequence alignment of PET-degrading enzymes. All enzymes involved in the tree generation in Fig. 6a were aligned. Key residues involved in the enzyme catalysis and the constitution of subsite I and subsite II are indicated by red-, blue-, and purple-colored triangles, respectively as same in Fig. 1a. At the left side, types of each enzymes are indicated.

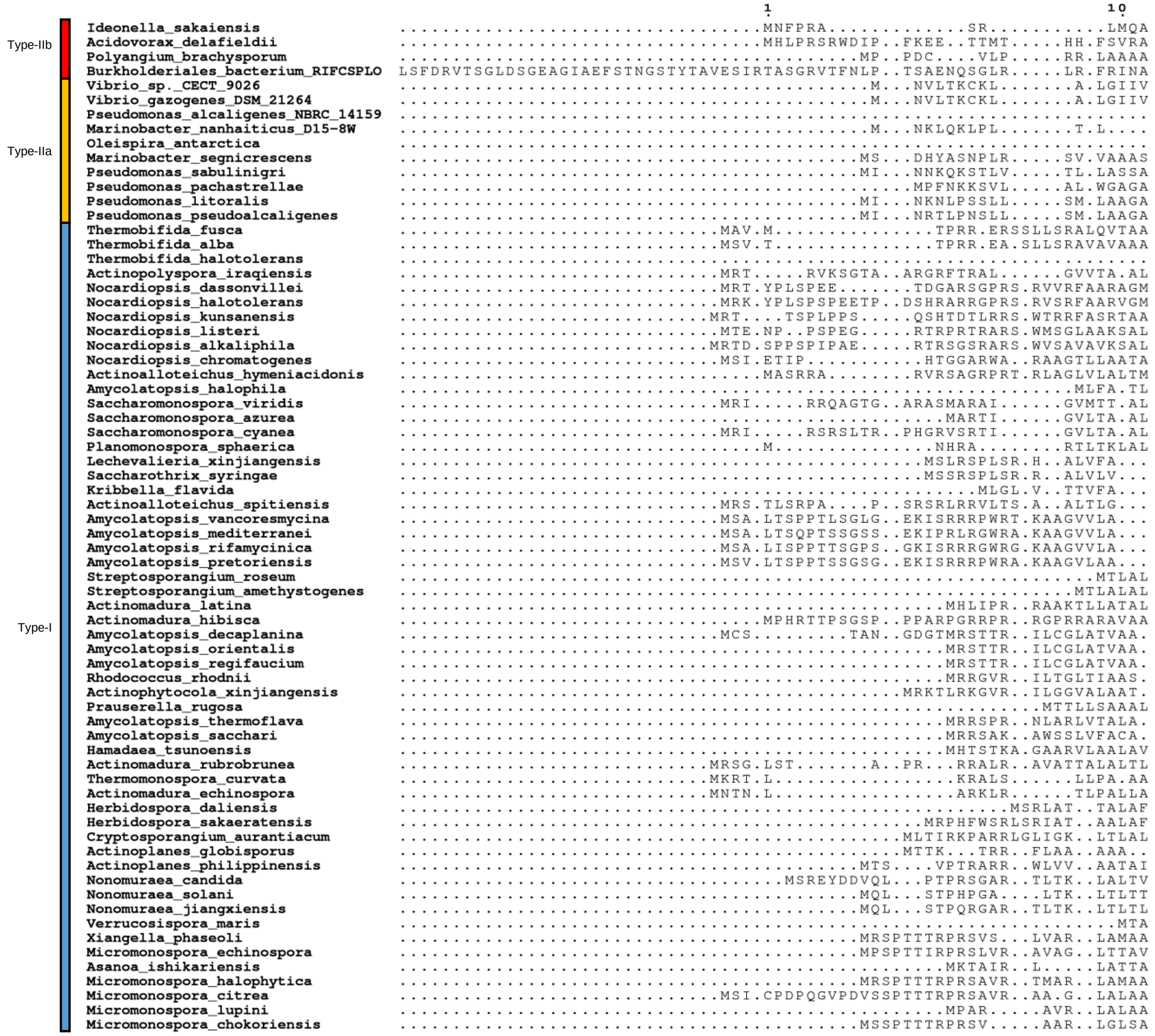

Supplementary Figure 5. Amino acid sequence alignment of PET-degrading enzymes (continued).

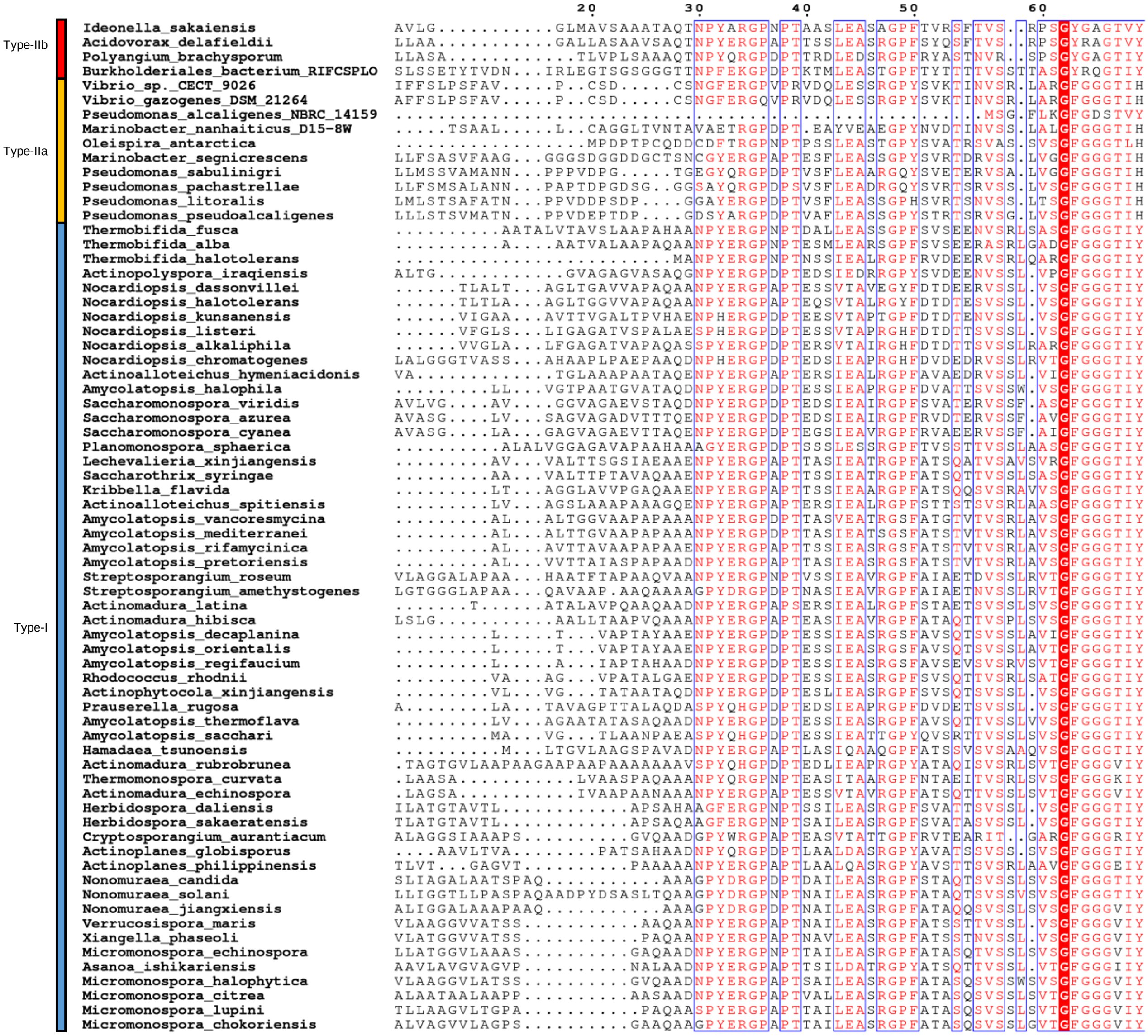

Supplementary Figure 5. Amino acid sequence alignment of PET-degrading enzymes (continued).

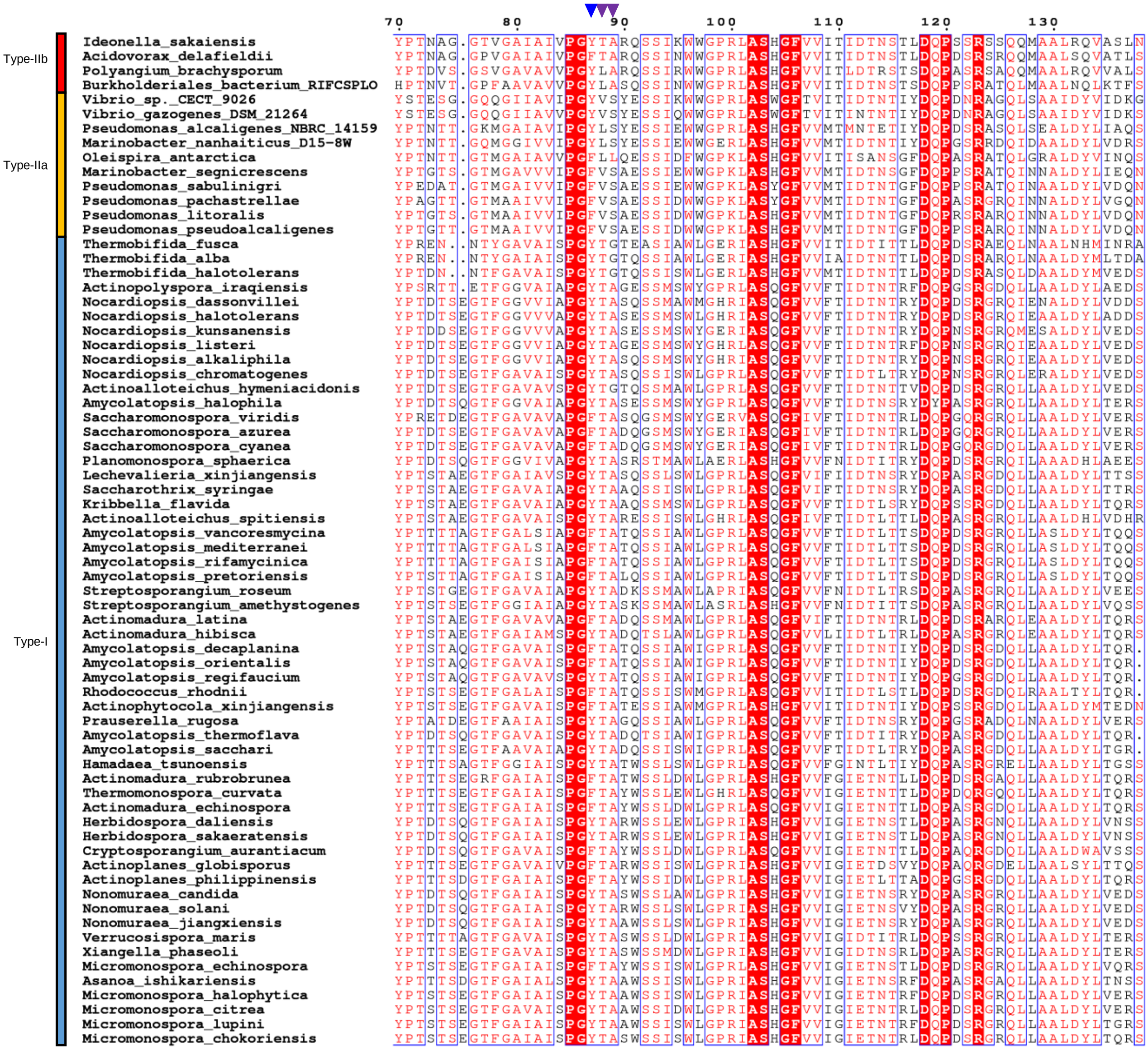

Supplementary Figure 5. Amino acid sequence alignment of PET-degrading enzymes (continued).

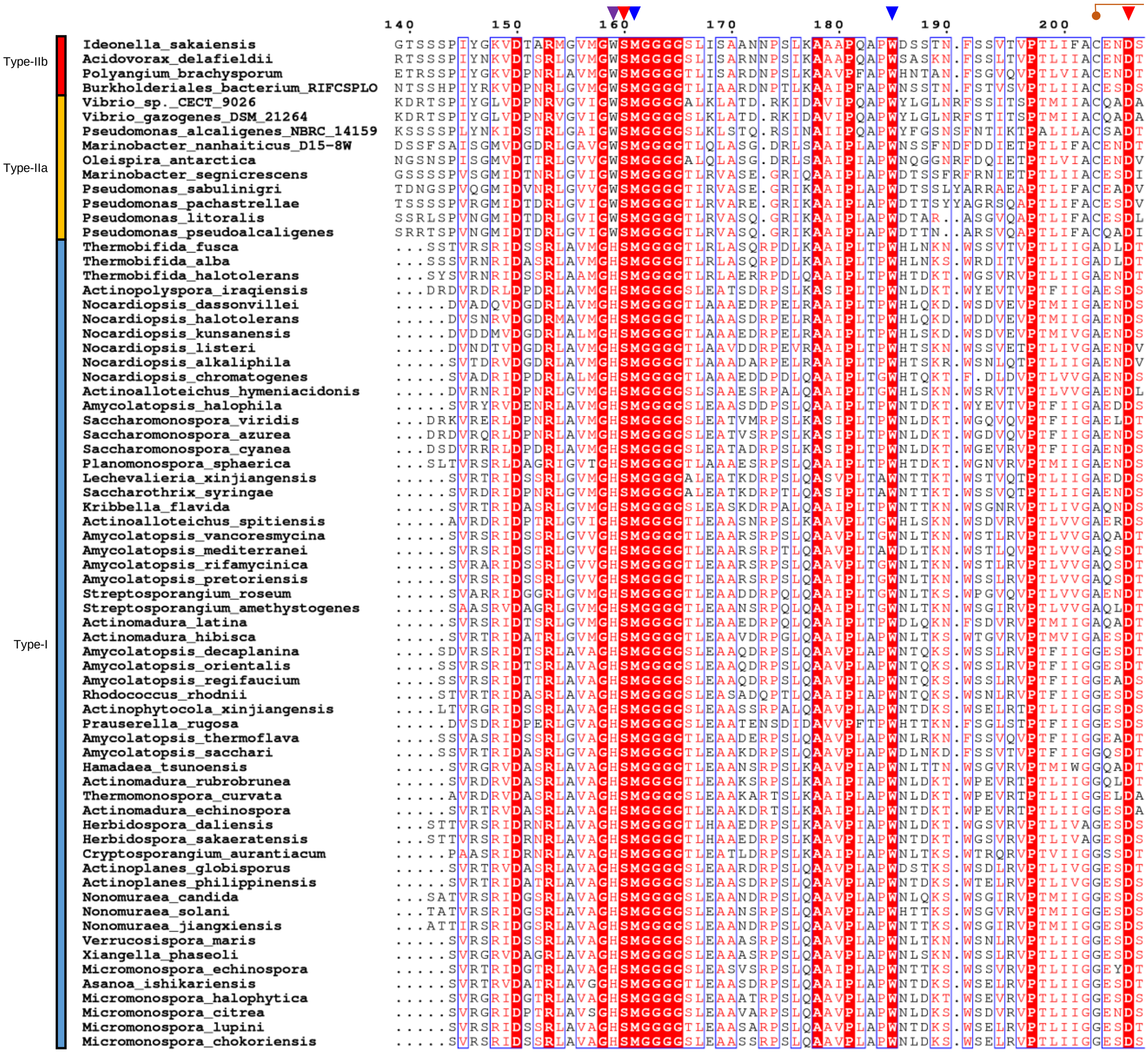

Supplementary Figure 5. Amino acid sequence alignment of PET-degrading enzymes (continued).

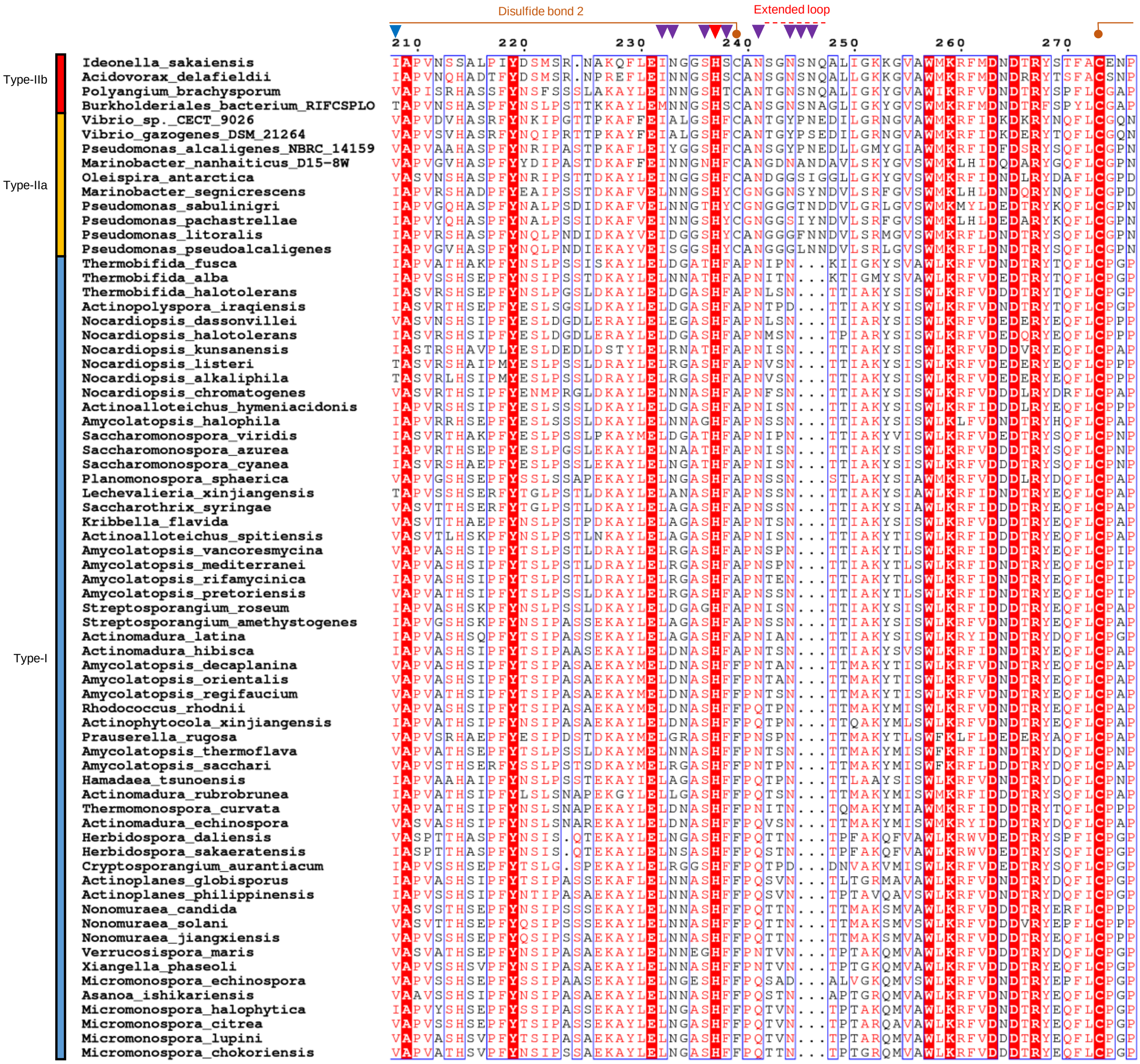

Supplementary Figure 5. Amino acid sequence alignment of PET-degrading enzymes (continued).

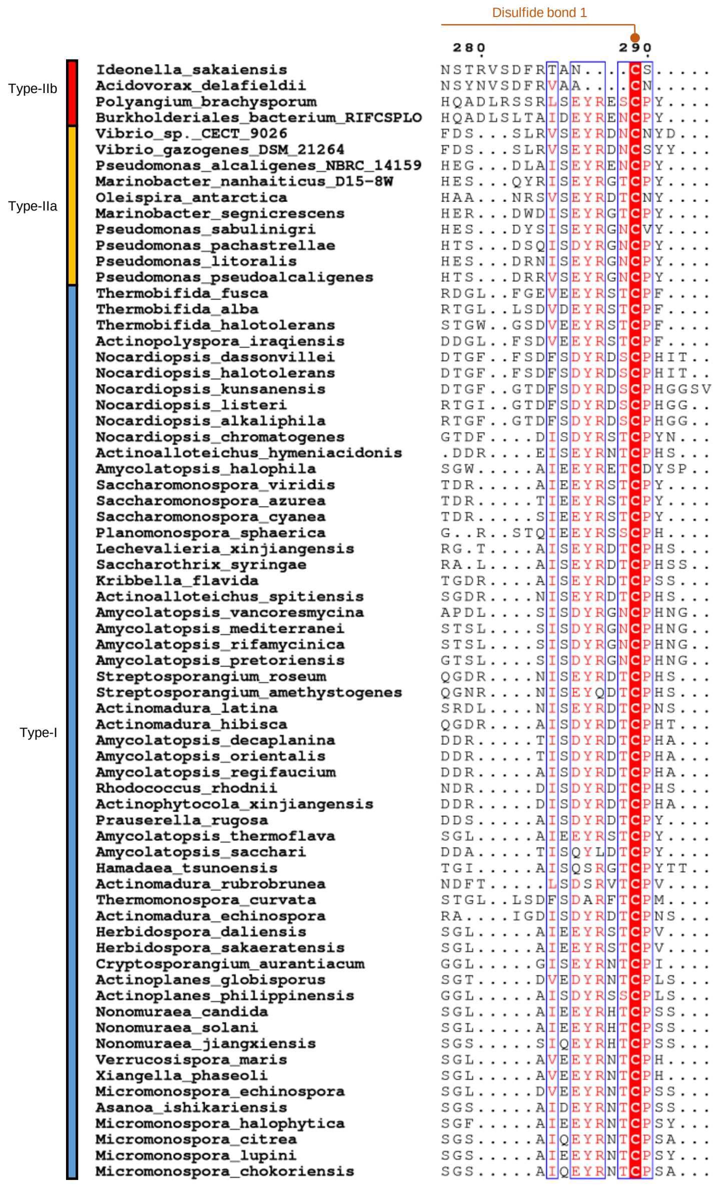

Supplementary Figure 5. Amino acid sequence alignment of PET-degrading enzymes.

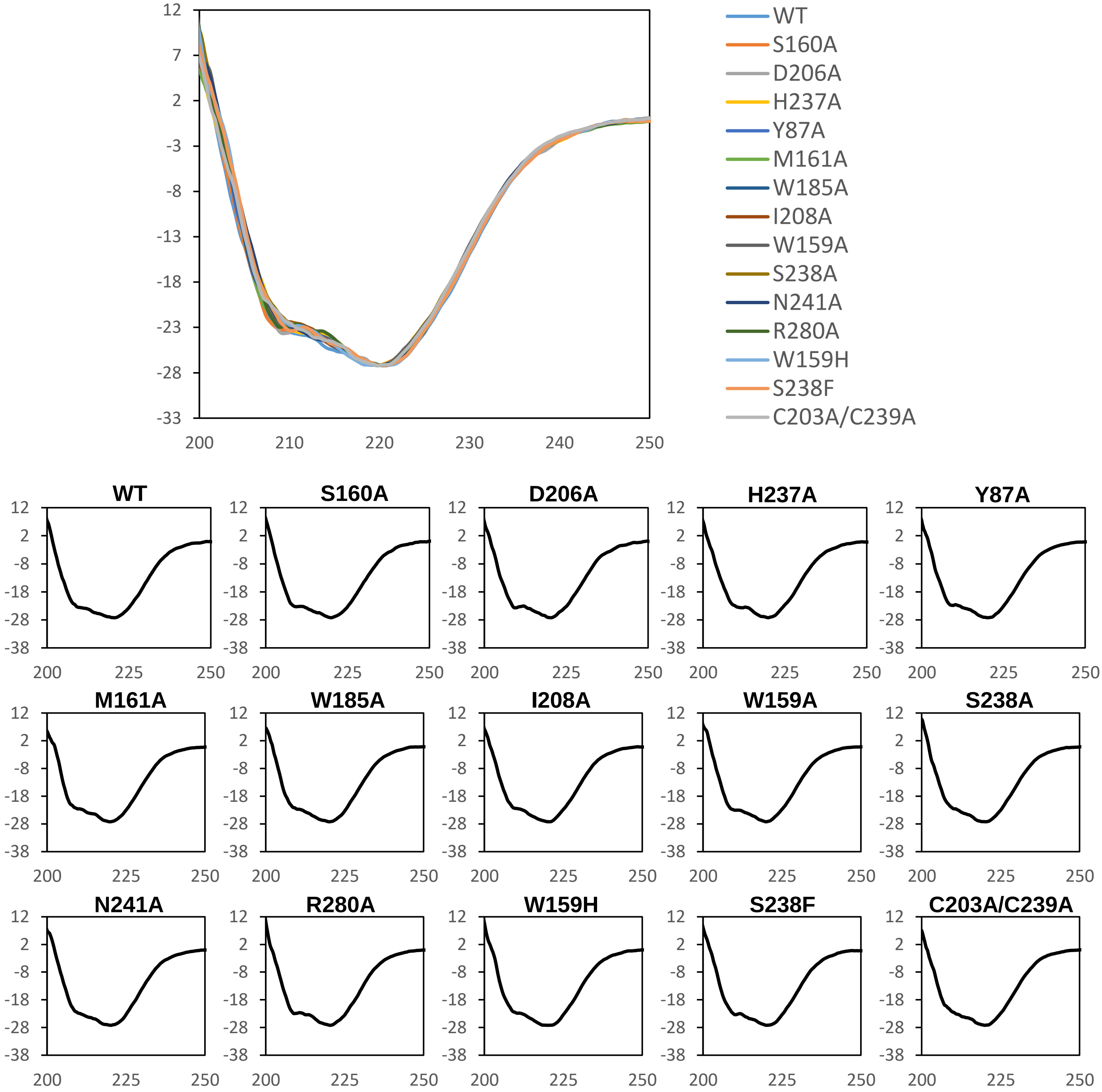

Supplementary Figure 6. Far-UV Circular dichroism. Above figure includes superposed CD spectra of the *Is*PETase variants used in this study. Each spectrum of the variants is also shown in below. The spectra indicate that there are no significant difference among the variants and *Is*PETase$^{W/T}$.

	*Is*PETase	*Is*PETase^{R280A}
**PDB code**	5XJH	5YNS
**Data collection**		
Wavelength (Å)	0.97934	0.97934
Unit cell (*a*, *b*, *c*; γ) (Å; °)	43.48, 50.40, 129.49; 90.0	43.61, 50.59, 129.58; 90.0
Space group	$P2_12_12_1$	$P2_12_12_1$
Solvent content (%)	53.38	52.66
Protein chains in AU	1	1
Resolution range (Å)	50.00-1.55	50.00-1.36
Highest resolution shell (Å)	1.58-1.55	1.38-1.36
Unique reflections	42939	62434
Redundancy	6.5(5.9)	9.3(5.7)
Completeness (%)	99.9(99.9)	98.7(96.9)
R_{merge} (%)	6.8(29.8)	6.7(30.1)
Average I/σ(I)	31.4(5.2)	40.8(4.6)
B from Wilson plot (Å^2)	14.2	10.8
**Refinement**		
R (%)	15.9	16.6
R_{free} (%)	19.2	19.2
Mean B value (Å^2)*	16.1	15.0
RMS deviation bond lengths (Å)	0.026	0.025
RMS deviation bond angles (°)	2.286	2.249
Number of amino acid residues	272	264
Number of water molecules	150	294
**Ramachandran plot**		
Most favored regions (%)	97.3	97.6
Additional allowed Regions (%)	2.7	2.4

*Mean B value is for both protein atoms and the solvent molecules.

Supplementary Table 1. Data collection and refinement statistics of *Is*PETase.

Extra amino acids at N-terminus	MGSSHHHHHHSSGLVPRGSHM
Codon optimized DNA sequence used for expression of *Is*PETase	CGCGGTCCGAATCCGACAGCCGCCAGTTTGGAAG<br>CGAGCGCTGGTCCATTCACCGTTCGCTCCTTTACC<br>GTGAGTAGACCGAGCGGTTATGGCGCTGGCACCG<br>TTTACTATCCAACAAATGCTGGGGGTACCGTGGGC<br>GCCATAGCCATAGTTCCCGGGTATACGGCACGGCA<br>GTCATCAATTAAATGGTGGGGACCGCGTCTGGCAT<br>CCCACGGTTTCGTAGTAATTACAATTGACACAAATT<br>CCACGTTAGACCAGCCATCAAGTCGGAGTTCGCAA<br>CAAATGGCCGCGCTGCGCCAGGTGGCGTCGTTAA<br>ACGGTACAAGTAGCAGCCCGATTTACGGAAAGGTC<br>GATACCGCTCGTATGGGTGTTATGGGGTGGAGTAT<br>GGGAGGTGGAGGCTCCCTGATCTCTGCTGCTAAC<br>AACCCTTCGCTGAAAGCAGCGGCGCCTCAAGCAC<br>CATGGGATTCTTCGACAAATTTTAGTTCTGTAACTG<br>TGCCCACGCTGATCTTCGCATGTGAAAACGATAGT<br>ATAGCCCCGGTCAACTCTTCAGCACTTCCTATCTAT<br>GATTCTATGTCACGCAACGCTAAGCAGTTTCTCGA<br>AATTAATGGTGGCTCACATTCCTGTGCGAATAGCG<br>GCAATTCTAACCAAGCATTAATCGGAAAAAAAGGC<br>GTTGCATGGATGAAACGTTTTATGGACAATGATACT<br>AGGTATTCTACTTTTGCCTGCGAGAACCCGAATAG<br>CACCAGAGTGTCTGATTTTCGTACAGCGAATTGCA<br>GC
Extra amino acids at C-terminus	LEDPAANKARKEAELAAATAEQ

Supplementary Table 2. Codon-optimized DNA sequence used for expression of *Is*PETase and extra amino acids at the N- and the C-termini.
